# Association of a New Measure of Obesity with Hypertension and Health-Related Quality of Life

**DOI:** 10.1371/journal.pone.0155399

**Published:** 2016-05-16

**Authors:** Wankyo Chung, Chun Gun Park, Ohk-Hyun Ryu

**Affiliations:** 1 College of Business, Hallym University, Chuncheon, South Korea; 2 Department of Mathematics, College of Natural Sciences, Kyonggi University, Suwon, South Korea; 3 Division of Endocrinology and Metabolism, Department of Internal Medicine, College of Medicine, Hallym University, Chuncheon, South Korea; The Chinese University of Hong Kong, HONG KONG

## Abstract

**Background:**

Despite its shortcomings, body mass index (BMI) has traditionally been used to define obesity. Another recently introduced obesity measure, A Body Shape Index (ABSI), has been introduced to focus on abdominal obesity, but its applicability remains limited. We analyzed the statistical properties of the ABSI and propose a modified ABSI, the z-score of the log-transformed ABSI (LBSIZ), to improve its applicability. We also examined the sensitivity of the newly introduced index in diagnosing obesity based on the percentage of body fat and its ability to predict hypertension and impaired health-related quality of life (HRQOL).

**Methods and Results:**

We transformed the ABSI to the LBSIZ in order to create a standard normalized obesity measure. All available data from the Korea National Health and Nutrition Examination Survey (KNHANES) (1998–2012) have shown BMI to be highly correlated with weight (r = 0.85 for women, r = 0.87 for men) and waist circumference (WC) (r = 0.86 for women, r = 0.85 for men), but the LBSIZ was found to be weakly correlated with weight (r = 0.001 for women, r = 0.0001 for men) and moderately correlated with WC (r = 0.51 for women, r = 0.52 for men). BMI showed an inverted U-shaped pattern when plotted against age, but a linear pattern was observed for the LBSIZ, indicating they are different kinds of obesity measures. Logistic regression showed that the odds ratio of obesity for the LBSIZ was 1.86 (95% confidence interval [CI] = 1.73–2.00) for males and 1.32 (95% CI = 1.24–1.40) for females after adjusting for weight, height, age, and year of participation in the KNHANES. While both BMI and the LBSIZ were significantly related to hypertension, the LBSIZ alone was significantly associated with impaired HRQOL.

**Conclusions:**

The LBSIZ is a standard normalized obesity measure independent of weight, height, and BMI. LBSIZ is a new measure of abdominal obesity with the ability to predict hypertension and impaired HRQOL, irrespective of BMI.

## Introduction

Overweight and obesity are responsible for 5% of deaths worldwide and are one of the top five leading global risks for mortality [1]. Obesity has also increased in prevalence in Korea, becoming one of the most important public health concerns [2]. Obesity is associated with a significant increase in mortality and a higher risk of many disorders, including diabetes mellitus, hypertension, dyslipidemia, heart disease, stroke, sleep apnea, and cancer[3–6]. Furthermore, these obesity-related pathologic conditions affect the health-related quality of life (HRQOL) of obese persons [7, 8].

Obesity is a state of excessive body fat accumulation, but it is very difficult to measure. Therefore, obesity has traditionally been defined by body mass index (BMI; defined as weight [kg] /height [m^2^]), a crude index of weight for a given height that has been widely used due to its simplicity. Recently, however, the shortcomings of BMI as a measure of obesity have been acknowledged. It does not distinguish between muscle and fat, is inaccurate in predicting the percentage of body fat (PBF) [9], and is not a good measure for the risk of heart attack, stroke, or death [10–12].

The quest for a reliable and practical obesity index has begun to focus on abdominal obesity, for which waist circumference (WC) has been used as a measure complementary to BMI. Recently, A Body Shape Index (ABSI) was proposed to standardize WC according to weight and height. It has initially been shown to be more closely associated with the mortality of adults than BMI or WC in the United States, but it has also been shown to be negatively associated with blood pressure in a study of Portuguese adolescents and less strongly associated with hypertension than WC or BMI in a study of Indonesian adults [13–15].

We thus analyzed the statistical properties of the ABSI and proposed a modified ABSI, the z-score of the log-transformed ABSI (LBSIZ), to improve its applicability. We also examined the sensitivity of the newly introduced index in diagnosing obesity based on the PBF and its ability to predict hypertension and HRQOL.

## Methods

### Description of the data

This study was conducted using the source data from the Korea National Health and Nutrition Examination Survey (KNHANES). The KNHANES is a cross-sectional, nationwide survey that has been approved by the Institutional Review Board of the Korea Centers for Disease Control and Prevention, and all participants in the survey provided written informed consent. All data from the KNHANES were obtained in a fully anonymized and de-identified manner and thus this study was exempt from the requirement of approval by Hallym University Institutional Review Board. This survey used a stratified, multistage, clustered probability sampling method to select a representative sample of the non-institutionalized civilian Korean population. It consisted of one health interview and three sub-surveys: (1) a health behavior survey, (2) a health examination survey, and (3) a nutrition survey [16].

The present study included adults at least 20 years of age from the KNHANES I (1998), II(2001), III(2005), IV(2007, 2008, 2009),and V(2010, 2011, 2012). On average, approximately 6,100 adults were sampled each year, ranging from 2,970(2007) to 7,893(1998). Pregnant women were excluded. The total number of participants in the analysis was 54,893.

Although the entire sample of 54,893 participants was used to analyze the statistical properties of the LBSIZ, the sample was further limited to the 18,615 adults for whom data on the PBF were available (2008–2011), in order to examine the sensitivity of the new index in diagnosing obesity. Their PBF was calculated from their body composition data, total fat mass, and total body mass, as measured by dual-energy X-ray absorptiometry (DXA) using a Discovery-W fan beam densitometer (Hologic, Bedford, MA, USA)according to standard procedures. The stability of the DXA measurements was determined by daily calibration with a phantom supplied by the manufacturer. The height was determined to the nearest 0.1 cm with a wall-mounted stadiometer. Weight was measured with light clothing but without shoes to the nearest 0.1kg, and waist circumference was taken at the midpoint between the lower border of the rib cage and the iliac crest to the nearest 0.1cm.

Furthermore, the limited sample also provided data on the EuroQOL-5 dimension (EQ-5D) index and the EuroQOL-visual analogue scale (EQVAS)developed by the EuroQOL group to measure HRQOL. The EQ-5D consists of five questions evaluating the level of self-reported problems in five dimensions (mobility, self-care, usual activities, pain or discomfort, and depression or anxiety), with three possible answers for each item (1, no problem; 2, moderate problem; 3, severe problem). A summary index(the EQ-5D index), calculated using a combination of the score of each of the five dimensions, ranges from −0.171 to 1, where the maximum score of 1refers to the best possible health status with no problems in any of the five dimensions. Participants also described their subjective health status for the EQ-VAS, with answers ranging from 0 (the worst imaginable health status) to 100(the best imaginable health status) [17].

### Measures of obesity

BMI (weight [kg]/height [m^2^]) is based on the log-log regression [ln(*weight*) = *b*_*0*_+ *b*_*1*_ln(*heigh*t)+*δ*], where the exponent *b*_*1*_ can be estimated to be 2. Although the estimated exponent ranges from 1.92 to 1.96 for U.S. males and from 1.45 to 1.95 for U.S. females, 2 is conventionally used for its simplicity [18, 19].

In the same vein, ABSI [(WC)/(weight^a1^∙height^a2^)] has been developed based on another log-log regression:

$$\text{log}(wc)=a_{0}+a_{1}\text{log}(w)+a_{2}\text{log}(h)+\varepsilon =a_{0}+a_{1}\text{log}(BMI)+(2a_{1}+a_{2})\text{log}(h)+\varepsilon ,$$ (1)

where *ε* is a normally distributed random variable with a mean of zero and a constant variance. While BMI standardizes weight for height, ABSI standardizes waist circumference for both weight and height, making ABSI uncorrelated with weight, height, and therefore BMI. ABSI is more focused on abdominal obesity, controlling for the confounding effects of weight and height. Using data on a sample of U.S. adults, Krakauer and Krakauer [13] estimated *a*_*1*_ as 0.681 and *a*_*2*_ as −0.814 and approximated them to 2/3 and −5/6, respectively, resulting in ABSI (WC/(weight^2/3^∙height^−5/6^).

In practice, as the estimated *b*_*1*_ for BMI varies by gender and country [18], the estimated values of *a*_*1*_ and *a*_*2*_ for ABSI also vary by age, gender, and country. For example, Cheung [15] found the values of *a*_*1*_ (0.632) and *a*_*2*_ (−0.801) for Indonesian adults, with a statistically significant difference by gender.

Eq 1can be transformed into the following:

$$ABSI=\frac{wc}{w^{a_{1}}h^{a_{2}}}=e^{a_{0}}e^{\varepsilon }.$$ (2)

Note that ABSI is an exponential function with base *e* and the powers *a*_*0*_ and *ε* in Eq 2 and may be skewed. Therefore, log-transformation of the ABSI can make it symmetric. Mathematically, the log-transformed ABSI (LBSI) is

$$LBSI=\text{log}(ABSI)=a_{0}+\varepsilon .$$ (3)

The LBSI is not only normally distributed with regard to the mean (*a*_*0*_) but is also symmetric when *ε* is assumed to be a normally distributed random variable with a mean of zero and a constant variance.

The LBSI can then be standardized by the z-score to remove the mean (*a*_*0*_). The z-score is the most frequently used way to compare statistics across any divided groups. We preferred the LBSI to the ABSI to standardize using the z-score because the LBSI is more likely to be normally distributed or symmetric, and has a mean of zero and a standard deviation of one when standardized. Therefore, the z-score for the LBSI (LBSIZ) is a standard normal distribution (or a symmetric distribution), and is defined as

$$LBSIZ=\frac{LBSI-LBSI(mean)}{LBSI(s.d.)}=\frac{\varepsilon -\varepsilon (mean)}{\varepsilon (s.d.)}.$$ (4)

Clearly, the LBSIZ has the additional advantage of being able to serve as a standard normalization for ABSI across any divided group by age, gender, race, or country, by simply including those variables in the estimation of Eq 1 and using their remaining residual ε for standard normalization.

## Results

The descriptive statistics of the 54,893 participants included in the study are as follows. The mean age was 48.7years (range, 20–103 years) and 56.7% were female. The mean waist circumference was 0.81 m (standard deviation [SD], 0.10 m), the mean height was1.62 m (SD, 0.09 m), and the mean weight was 61.88 kg (SD, 11.26 kg).

Table 1 shows the coefficients resulting from log-log regression, which are the scaling exponents used to calculate ABSI. The estimated coefficients for log(weight) and log(height) in Korea were different from those in the US. The scaling exponents to calculate (American) ABSI, 0.6807 and −0.814, were out of the corresponding 95% confidence intervals in Korea. They also differed significantly across gender (P<0.001).Due to the presence of gender differences, separate coefficients were estimated and used to generate the LBSIZ.

**Table 1:** Regression coefficients of the relationships between the log of waist circumference (m) and the log of weight (kg) and the log of height (m).

Variables	U.S.	Korea		
		All (54,893)	Men (23,767)	Women (31,126)
Intercept	−2.589 (0.020)	−2.6911* (0.0065)	−2.4072* (0.0097)	−2.5940* (0.0095)
Log(weight)	0.6807 (0.0052)	0.7264* (0.0019)	0.6724* (0.0028)	0.7374* (0.0025)
Log(height)	−0.8140 (0.020)	−1.0633* (0.006)	−1.1457* (0.011)	−1.4053* (0.0093)
R^2^	0.83	0.73	0.72	0.73

() is standard error. See Eq 1.

Coefficients for the U.S. were taken from Krakauer and Krakauer (2012).

*p<0.001.

Table 2 presents the correlation coefficients of BMI and the LBSIZ with anthropometric measures. Because men and women are different in body shape and size, the coefficients are presented separately, with those above the diagonal for women and those below the diagonal for men. BMI was highly correlated with weight (r = 0.85 for women, r = 0.87 for men) and WC(r = 0.86 for women, r = 0.85 for men), while the LBSIZ was weakly correlated only with WC(r = 0.51 for women, r = 0.52 for men). It should be noted that BMI itself was weakly and not statistically significantly correlated with the LBSIZ (r = −0.003 for women, r = −0.002 for men), indicating that they are different kinds of obesity measures.

**Table 2:** Correlation coefficients between obesity measures and anthropometric measures by sex.

	BMI	LBSIZ	Weight	WC	Height
BMI	1	−0.0028	0.8500*	0.8555*	−0.1626*
LBSIZ	−0.0024	1	0.0013	0.5105*	0.0005
Weight	0.8669*	0.0001	1	0.7407*	0.3753*
WC	0.8454*	0.5229*	0.7711*	1	−0.1182*
Height	0.0630*	−0.0009	0.5468*	0.1276*	1

Figures above the diagonal correspond to females and those below the diagonal refer to males.

*p<0.01.

BMI, body mass index; LBSIZ, z-score of the log-transformed A Body Shape Index; WC, waist circumference.

**Fig 1:**
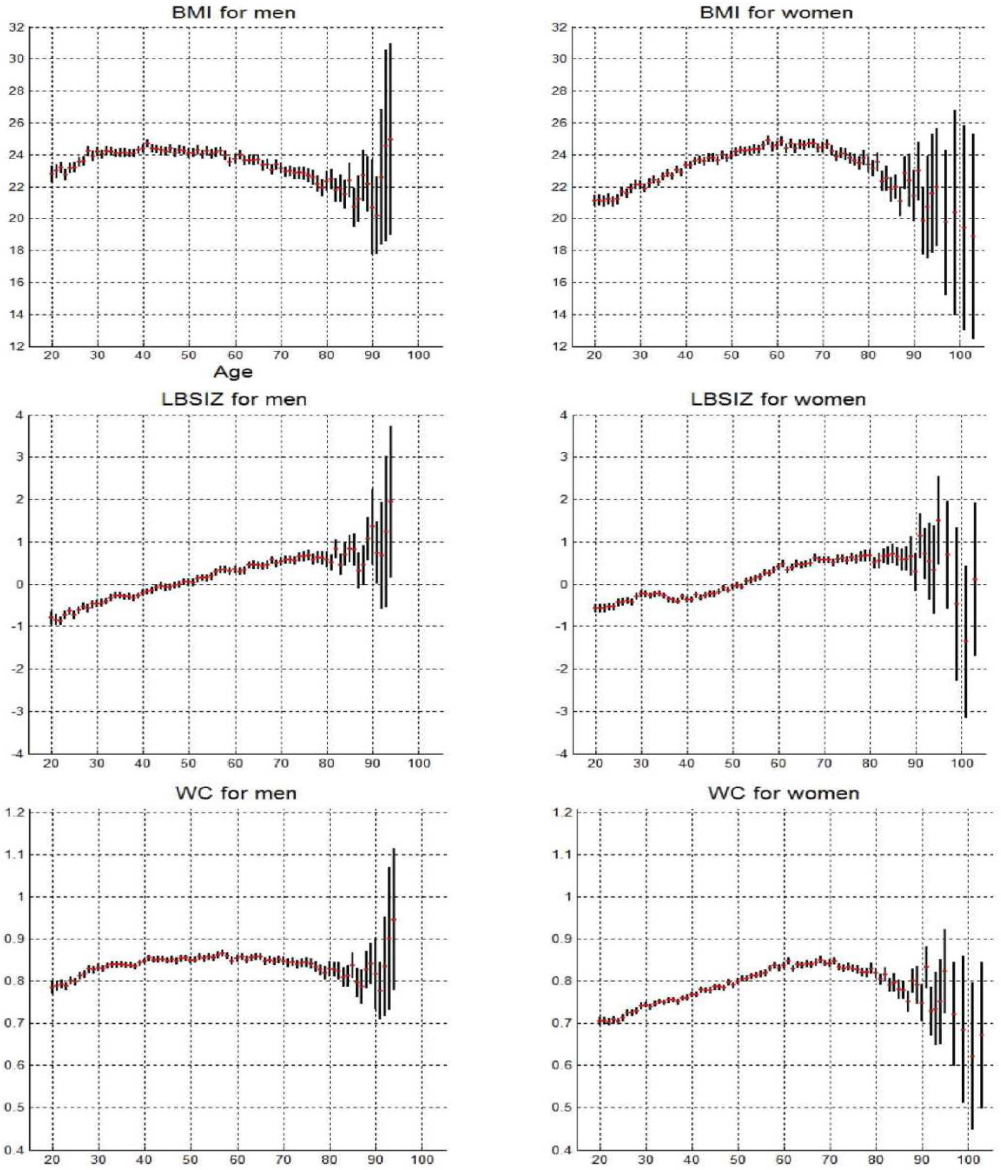
Mean values of body mass index (BMI), z-score of the log-transformed A Body Shape Index (LBSIZ), and waist circumference (WC) with increasing age.

Further differences between BMI and the LBSIZ are shown in Fig 1, which examines how their mean values changed with age. While BMI showed an inverted U-shaped pattern, increasing and then decreasing with age, LBSIZ increased consistently with age. The fact that even WC showed an inverted U-shaped pattern with age indicates that the LBSIZ is an obesity measure that is different from not only BMI, but also WC itself, as it standardizes WC for weight and height.

We then examined the sensitivity of the LBSIZ in diagnosing obesity based on the PBF. From the sample of 54,893 participants, we analyzed the 18,615 adults for whom data on the PBF were available (2008–2011). Using this smaller sample, the estimated coefficient *a*_*1*_ changed from 0.7264 to 0.7438 and *a*_*2*_changed from −1.0633 to −1.1082. We used both the continuous measure of PBF and the World Health Organization’s standard for obesity (PBF>25% for males and >30% for females) as dependent variables [20].

At a given height and weight, the LBSIZ was positively associated with the PBF [coefficient 1.52 (standard error, 0.05) for males and for females [0.60 (standard error, 0.04)] after adjusting for age and year of participation in the KNHANES. Logistic regression demonstrated that the odds ratio of obesity for the LBSIZ was 1.86 (95% confidence interval [CI] = 1.73–2.00) for males and 1.32(95% CI = 1.24–1.40)for females after adjusting for weight, height, age, and year of KNHANES participation (Table 3). Interestingly, the odds ratio of obesity for BMI was 1.46 (95% CI = 1.13–1.90) for males and 2.70 (95% CI = 2.00–3.65) for females. While both the LBSIZ and BMI were associated with PBF, the LBSIZ appeared to be more closely associated with PBF in males and BMI with PBF in females. Moreover, due to the high correlation between BMI and weight, weight became insignificant (P = 0.812 for males) when used together with BMI, while it remained significant when used together with the LBSIZ.

**Table 3:** Odds ratios of obesity defined by percentage of body fat (>25% for males, >30% for females).

	Males (n = 7,972)		Females (n = 10,643)	
BMI	1.46* (1.13–1.90)		2.70* (2.00–3.65)	
LBSIZ		1.86* (1.73–2.00)		1.32* (1.24–1.40)
Weight	1.01 (0.92–1.11)	1.16* (1.15–1.17)	0.85* (0.75–0.96)	1.28* (1.27–1.30)
Height	0.39 (0.00–776.40)	0.00* (0.00–0.00)	30658.75 (8.15–115000000)	0.00* (0.00–0.00)
Age	1.02* (1.01–1.02)	0.99* (0.99–1.00)	1.00 (0.99–1.00)	0.99* (0.98–0.99)

() is the 95% confidence interval. Values were adjusted for year participation in the Korea National Health and Nutrition Examination Survey (2008, 2009, 2010, and 2011).

*p<0.01.

BMI, body mass index, LBSIZ, z-score of log-transformed A Body Shape Index.

Next, we examined the ability of both measures of obesity to predict other outcomes: a measure of morbidity and impaired quality of life. We used hypertension (defined as systolic blood pressure ≥140mmHg, diastolic blood pressure≥90mmHg, or the use of anti-hypertensive drugs) as a measure of morbidity and the lowest quartiles of the EQ-5D index and the EQ-VAS as a measure of impaired quality of life. The results presented in Table 4 show that both BMI and the LBSIZ were significantly related to hypertension. However, the LBSIZ alone was significantly associated with impaired quality of life. The multivariate odds ratios of the LBSIZ for impaired quality of life were 1.27 for the EQ-5D index(95% CI = 1.18–1.36) and 1.20 for the EQ-VAS (95% CI = 1.13–1.28) for males, and 1.16 for the EQ-5D index(95% CI = 1.10–1.22) and 1.11 for the EQ-VAS (95% CI = 1.05–1.16) for females after adjusting for weight, height, age, and year of KNHANES participation.

**Table 4:** Odds ratios of hypertension and health-related quality of life.

	Hypertension		Health-related quality of life			
			EQ-5D Index(first quartile)		EQ-VAS(first quartile)	
	Male	Female	Male	Female	Male	Female
BMI	1.71* (1.34–2.18)	1.39* (1.15–1.68)	1.16 (0.90–1.49)	1.37* (1.17–1.61)	1.02 (0.83–1.27)	0.96 (0.83–1.11)
LBSIZ	1.17* (1.09–1.26)	1.22* (1.14–1.30)	1.27* (1.18–1.36)	1.16* (1.10–1.22)	1.20* (1.13–1.28)	1.11* (1.05–1.16)
n	7,963	10,631	7,912	10,562	7,897	10,513

() is the 95% confidence interval. Values were adjusted for weight, height, age, and year of participation in the Korea National Health and Nutrition Examination Survey (2008, 2009, 2010, and 2011).

*p<0.01.

BMI, body mass index; LBSIZ, z-score of log-transformed A Body Shape Index.

## Discussion

Despite its shortcomings, BMI has been conventionally used as a measure of obesity, largely due to its simplicity[7].BMI does not distinguish between muscle and fat and cannot discriminate fat location[21].Fat distribution may be more important than the total quantity of body fat in predicting the risk of metabolic disease [22, 23]. Even in a study of Koreans with a normal BMI, strong and graded associations were found between increased PBF and the prevalence of CVD risk factors [24]. Meanwhile, WC is strongly related to visceral fat depots [25]. Therefore, WC is now widely used to measure abdominal obesity.

However, WC has limitations in its use due to high collinearity with weight and BMI, and thus its role in complementing BMI in predicting mortality and health risks is limited. Recently, ABSI has been developed to focus on abdominal obesity, standardizing waist circumference for height and weight [13].ABSI measures waist circumference in relation to weight and height and thus can be a measure of abdominal obesity independent of weight, height, or BMI.

Nonetheless, ABSI seems to lack the statistical sophistication to be widely used clinically and in public health. As an alternative to ABSI, the z-score of the log-transformed ABSI (LBSIZ) has a standard normal distribution. Moreover, to improve its generalizability and applicability across groups of people depending on national origin or gender, we can simply include any categorical variables for age, gender, and/or race in Eq 1and calculate the LBSIZ using the remaining residual ε for standard normalization according to Eq 4.

Based on the data from a large-scale, nationally representative population survey, the exponents used to generate ABSI were found to differ between Korean and U.S. subjects and between Korean males and females. Cheung [15] found similar results, necessitating different exponents in the U.S. and Indonesia to calculate ABSI. Moreover, the exponents were found to be age-dependent [26]. The LBSIZ overcomes this limitation of ABSI to create an obesity measure normalized by country, gender, and/or age. Moreover, the standard normal distribution of the LBSIZ may help to solve the arbitrary choice of cutoff points in defining obesity based on BMI and WC across ethnicities, countries, and genders [27, 28]. LBSIZ can define relative obesity (e.g. the upper 5% of obesity corresponding to an LBSIZ value >1.65) rather than absolute obesity (e.g., obese or not).

Meanwhile, the correlation coefficients and the graphical examination of the data showed that the LBSIZ measures abdominal obesity independently of weight, height, or BMI. The clear differences between the LBSIZ and BMI can be seen when they are graphed according to age. While BMI and even WC showed an inverted U-shaped pattern, increasing and then decreasing with age, LBSIZ increased over the entire course of the lifespan. Furthermore, while both the LBSIZ and BMI are associated with PBF, the former appears to be more closely associated with male PBF and the latter with female PBF. Additionally, unlike BMI, the LBSIZ alone is useful in defining obesity independently of weight, due to their limited correlation with each other.

Krakauer and Krakauer [13] found a positive association of ABSI with U.S. mortality and Cheung [15] identified a positive association of ABSI with hypertension in Indonesia, although this association was weaker than that of BMI. Duncan [14], however, showed a negative association of ABSI with blood pressure in Portuguese adolescents. In our study, both BMI and the LBSIZ were significantly related to hypertension. Meanwhile, impaired HRQOL, especially in the domains of physical function, has been found for obese individuals as measured by BMI [7, 8].In more recent studies, abdominal obesity was shown to be associated with lower quality of life [29–31].However, the LBSIZ alone was significantly associated with impaired HRQOL in our study. Therefore, the LBSIZ is a measure of abdominal obesity with the ability to predict both an outcome of morbidity and impaired quality of life, regardless of BMI. Notably, ABSI is highly correlated with the LBSIZ (r = 0.96) but when ABSI (or WC) was used directly in our logistic estimations of PBF, hypertension, and impaired quality of life, it inflated the respective coefficients beyond any reasonable interpretation.

Our study has some limitations. First, the newly proposed LBSIZ measure needs to be examined in the future to determine how closely it is associated with mortality and other morbidities. Second, hip circumference and related measures of obesity, such as waist-to-hip ratio or body adiposity index(hip circumference/height^1.5^),could not be evaluated in this study because hip circumference was not available in the data we used [32,33].

The need for useful public health and clinical obesity measures has increased overtime. The accumulating evidence on abdominal obesity sheds light on the potential role of an improved measure, such as the LBSIZ. While BMI may have been used too long for its shortcomings to be corrected, ABSI is new enough that another modified measure, such as the LBSIZ, could be developed. How well it complements BMI in predicting obesity, morbidity, and mortality remains to be seen.
